# Supplementary material for: A Global Screen for Assembly State Changes of the Mitotic Proteome by SEC-SWATH-MS
Source: Cell Syst. 2020 Feb 26;10(2):133–155.e6. doi: 10.1016/j.cels.2020.01.001 (PMC7042714; doi:10.1016/j.cels.2020.01.001)

Q70E73 | RAPH1_HUMAN | RAPH1 ALS2CR18 ALS2CR9 KIAA1681 LPD PREL2 RMO1
Monomer MW [kDa]: 135.256 Monomer expected elution fraction: 38

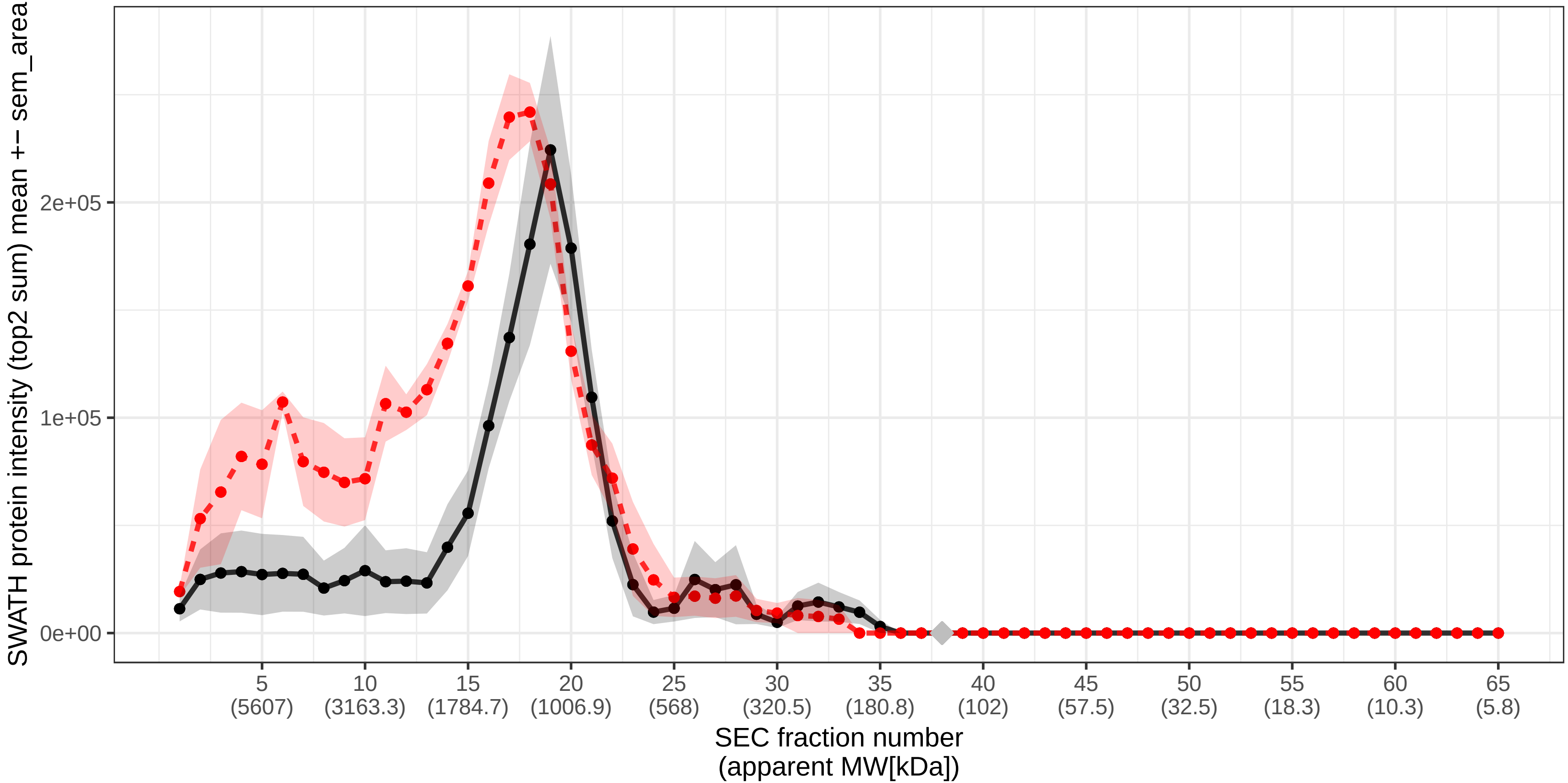

Supplement: Data S1. SEC-SWATH-MS Protein Chromatograms, Related to Figure 1 [file mmc6.zip › SECchrom_Q70E73_RAPH1_HUMAN_RAPH1_ALS2CR18_ALS2CR9_KIAA1681.pdf]
